# Breeding triple‐advantage cottonseed with higher yield, enhanced nutrition, and reduced toxicity by redirecting terpenoid metabolism to astaxanthin

**DOI:** 10.1111/pbi.70116

**Published:** 2025-04-29

**Authors:** Lu Long, Ying‐Chao Tang, Zhen‐Nan Zhang, Yi‐Bo Fan, Guan‐Ying Wang, Ting‐Wan Li, Gai‐Yuan Hu, Shen‐Zhai Shang, Man Jiang, Hao‐Ge Song, Yuan‐Mei Miao, Zhong‐Ping Xu, Shuang‐Xia Jin, Wei Gao

**Affiliations:** ^1^ National Key Laboratory of Cotton Bio‐breeding and Integrated Utilization, School of Life Science Henan University Kaifeng Henan China; ^2^ Hubei Hongshan Laboratory, National Key Laboratory of Crop Genetic Improvement Huazhong Agricultural University Wuhan Hubei China

**Keywords:** astaxanthin, *CrBKT*, metabolic engineering, synthetic biology, gossypol, cottonseed improvement

## Abstract

Cottonseed is a valuable source of edible oil and protein, but its utilization is limited by high gossypol content. In this study, we engineered cotton (*Gossypium hirsutum*) to biosynthesize astaxanthin through both single‐gene (*CrBKT*) and multi‐gene (*CrBKT*, *ZmPSY1*, *PaCrtI*, *HpCrtZ*) expression strategies. Transgenic cotton plants exhibited significant astaxanthin accumulation across multiple tissues, with distinct red pigmentation observed in leaves, stems, reproductive organs, and cottonseeds. While single *CrBKT* expression was sufficient to redirect metabolic flux toward astaxanthin biosynthesis, multi‐gene transformation did not necessarily lead to higher astaxanthin levels, suggesting that BKT is the key determinant of astaxanthin accumulation in cotton. Additionally, BKT‐overexpressing plants produced larger cottonseeds, with increased seed weight and size, indicating a possible link between carotenoid metabolism and seed development. Importantly, gossypol content was significantly reduced in transgenic cottonseeds, likely due to the redistribution of terpene metabolism. The qRT‐PCR analyses confirmed that the expression of key gossypol biosynthetic genes was downregulated, supporting a metabolic trade‐off between astaxanthin and gossypol biosynthesis. These results demonstrate that cotton can serve as a biofactory for astaxanthin production, providing a scalable and cost‐effective alternative to traditional sources. Furthermore, the dual benefits of enhanced nutrition and reduced toxicity significantly expand the potential applications of cottonseed in human food, animal feed, and functional ingredient markets.

## Introduction

Cotton fibre is an essential commodity in daily life and is often referred to as ‘white gold’. In addition to its fibre, cottonseed is a valuable byproduct, rich in unsaturated fatty acids, proteins, and essential minerals, making it highly nutritious and economically significant (Alford *et al*., 1996; Kumar *et al*., 2023; Yang *et al*., 2017). However, the presence of gossypol and its derivatives in cottonseed pigment glands‐classified as sesquiterpenes poses toxicity risks to humans and monogastric animals (Gao *et al*., 2020; Sunilkumar *et al*., 2006). Consequently, the application of cottonseed is largely restricted to ruminant feed and fungal culture media. Furthermore, the removal of gossypol during cottonseed oil extraction increases processing costs (Kumar *et al*., 2023). With the growing interest in edible cottonseed, research has increasingly focused on detoxification strategies, enhancing its nutritional value, and improving the antioxidant capacity of cottonseed oil (Li *et al*., 2024; Riaz *et al*., 2021). Enhancing its nutrient profile and economic value represents a promising direction for future cottonseed utilization.

Astaxanthin, a ketocarotenoid belonging to the terpene family, is a potent antioxidant with a unique structure comprising 13 conjugated double bonds and alternating polar–nonpolar–polar regions (Ambati *et al*., 2014). Astaxanthin also contains two β‐ionone rings with hydroxyl and keto functional groups, which enable astaxanthin to scavenge free radicals, giving it significantly higher antioxidant activity than vitamin E and coenzyme Q10 (Fang *et al*., 2019; Matsuno and Miki, 1990). Natural astaxanthin has been shown to reduce oxidative damage caused by free radicals and prevent the oxidation of unsaturated fatty acids (McNulty *et al*., 2007; Wang *et al*., 2019). These properties contribute to its pharmacological benefits against cancer, cardiovascular diseases, diabetes, and inflammation (Fang *et al*., 2019; Hussein *et al*., 2006). Additionally, astaxanthin has vast market potential in aquaculture, cosmetics, and dietary supplements (Shah *et al*., 2016).

Astaxanthin accumulates widely in marine organisms such as shrimp, crabs, and fish, but animals lack the ability to synthesize it de novo and must obtain it through their diet (Hussein *et al*., 2006; Routray *et al*., 2019). It contains three optical isomers: (3S, 3′S), (3R, 3′S), and (3R, 3′R) based on the position of chiral carbon atoms on β‐ionone rings (Fang *et al*., 2019). Currently, astaxanthin is primarily produced via chemical synthesis or extracted from *Haematococcus pluvialis* or marine wastes (Lorenz and Cysewski, 2000; Routray *et al*., 2019). Chemically synthesized astaxanthin consists of a mixture of these three isomers in a 1:2:1 ratio, whereas natural astaxanthin predominantly exists in the (3S, 3′S) and (3R, 3′R) forms. The antioxidant activity of synthetic astaxanthin is 20–50 times lower than that of its natural counterpart (Fang *et al*., 2019; Lorenz and Cysewski, 2000). As a result, using bacteria, algae, and plants as biological platforms for natural astaxanthin production is an emerging focus in industrial biotechnology.

Astaxanthin biosynthesis contains two stages: the formation of its direct carotenoid precursor, β‐carotene, followed by its conversion into astaxanthin. β‐Carotene synthesis originates from the precursors isopentenyl pyrophosphate (IPP) and dimethylallyl pyrophosphate (DMAPP), which can be synthesized via two distinct pathways: the mevalonate (MVA) pathway and the 2‐C‐methyl‐D‐erythritol 4‐phosphate (MEP) pathway (Vranová *et al*., 2013). Geranylgeranyl pyrophosphate (GGPP), the direct precursor of carotenoids, is synthesized from three molecules of IPP and one molecule of DMAPP via GGPP synthase (GGPPS). Phytoene synthase (PSY) catalyses the condensation of two GGPP molecules to produce phytoene, which is subsequently desaturated by carotenoid isomerase (CrtI) to form lycopene. Lycopene undergoes two major metabolic branches: one leading to α‐carotene and lutein, and the other forming β‐carotene via lycopene cyclase (LCY). The conversion of β‐carotene to astaxanthin varies significantly among species, requiring the introduction of hydroxyl (‐OH) and keto (‐C=O) groups at the β‐ionone rings. This transformation is catalysed by hydroxylases and ketolases, which convert β‐carotene into 3‐hydroxy‐4‐keto‐β‐rings, forming astaxanthin (Fang *et al*., 2019). The low efficiency of BKT (β‐carotene ketolase)‐mediated ketolation has been identified as a key limiting factor in astaxanthin accumulation in engineered plants (Zhong *et al*., 2011).

In nature, most higher plants, including cotton, lack carotenoid ketolase genes, rendering them incapable of astaxanthin biosynthesis. To overcome this limitation, researchers have introduced the β‐carotene ketolase gene *CrtO* from *H. pluvialis* into tobacco plastids, resulting in the accumulation of red (3S, 3′S) astaxanthin and other ketocarotenoids in the nectary tissues of transgenic tobacco plants (Mann *et al*., 2000). Since then, astaxanthin biosynthesis has been reconstructed in various crops, including *Arabidopsis* (Stålberg *et al*., 2003; Zhong *et al*., 2011), carrot (Ahn *et al*., 2012), tomato (Huang *et al*., 2013), apple (Jia *et al*., 2019), soybean (Pierce *et al*., 2015), maize (Liu *et al*., 2021; Zhu *et al*., 2008), and rice (Zhu *et al*., 2018), positioning these plants as potential dietary sources of astaxanthin.

In this study, we successfully established an astaxanthin biosynthetic pathway in cotton. Through testing multiple gene combinations, we discovered that the single‐gene expression of an exogenous *CrBKT* could drive high astaxanthin accumulation across various cotton tissues. The astaxanthin content in cotton leaves reached 126.17 mg/kg, positioning cotton as a promising host for natural astaxanthin production. Furthermore, astaxanthin accumulation in cottonseeds causes the metabolic trade‐off between astaxanthin and gossypol biosynthesis, resulting in larger cottonseeds with enhanced nutritional value (higher astaxanthin content), reduced toxicity (lower gossypol levels), and improved yield. These findings offer a novel strategy for cottonseed improvement, paving the way for enhanced utilization of cotton as a multipurpose crop.

## Results

### Design of a strategy for astaxanthin biosynthesis in cotton

**Figure 1:**
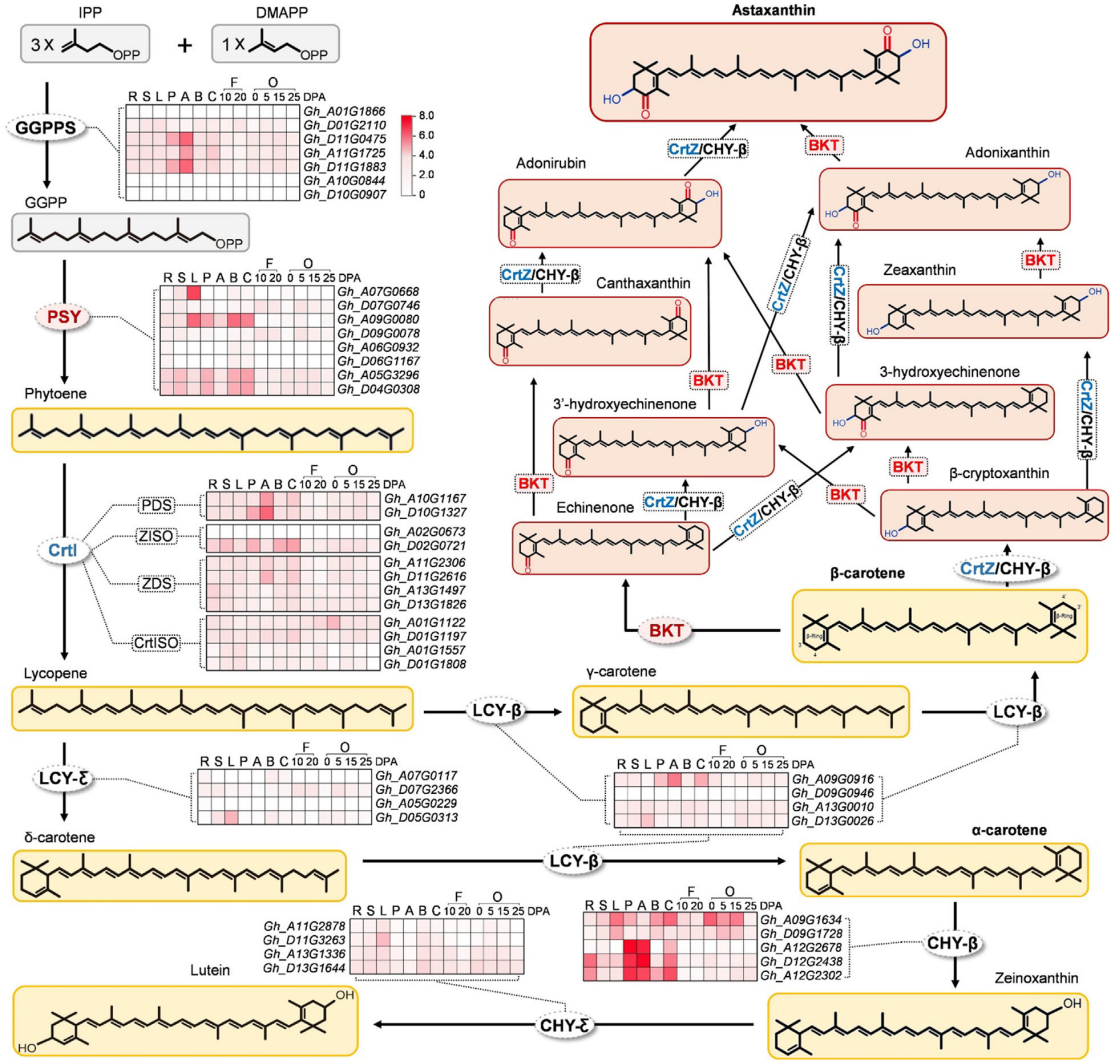
Carotenoid biosynthetic pathway and expression patterns of carotenoid biosynthetic genes in cotton. The heat map shows the expression patterns of genes in different organs. The RPKM values of each gene are represented by a gradient from low (white) to high (red). R, root; S, stem; L, leaf; P, petal; A, anther; B, bract; C, calyx; F, fibre; O, ovule; DPA, days post anthesis.

Based on previously reported carotenoid and astaxanthin biosynthetic pathways, we analysed publicly available cotton genome (Zhang *et al*., 2015) and transcriptome data (Yang *et al*., 2023) to assess the basal expression of carotenoid‐related genes. Figure 1 shows that homologues of all key enzymes involved in carotenoid biosynthesis were identified in the cotton genome, including GGPPS, which directs terpenoid precursors into the carotenoid metabolic pathway; PSY, the rate‐limiting enzyme in carotenoid biosynthesis, as well as downstream hydroxylase CHY‐β, CHY‐ε and others. Despite variations in gene expression levels across different tissues, the presence of these genes suggests that cotton possesses the necessary metabolic framework and precursor supply to support astaxanthin biosynthesis.

**Figure 2:**
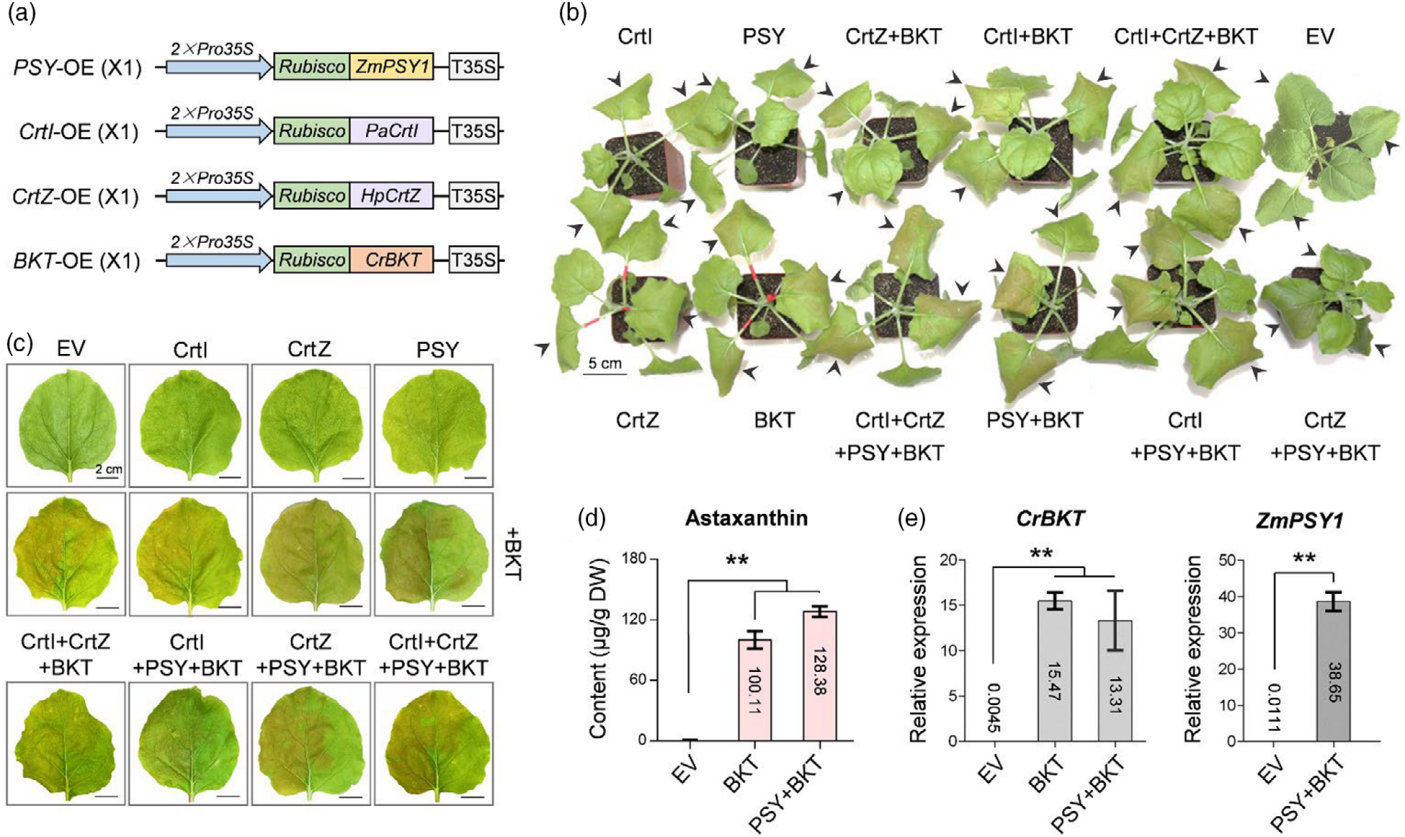
Transient expression of exogenous genes in tobacco leaves induces astaxanthin accumulation. (a) Schematic representation of single‐gene overexpression vectors (X1) used for transient and stable transformation. Genes (*ZmPSY1*, *PaCrtI*, *HpCrtZ*, or *CrBKT*) were driven by the enhanced 35S promoter for carotenoid and astaxanthin biosynthesis. (b) Representative tobacco leaves expressing single or multiple carotenoid and astaxanthin biosynthetic genes through *Agrobacterium*‐mediated transient expression. Scale bar, 5 cm. The black arrow indicates the infiltrated area. EV, empty vector. (c) Close‐up images of tobacco leaves expressing carotenoid and astaxanthin biosynthetic genes. Scale bar, 2 cm. (d) HPLC analysis of astaxanthin content in tobacco leaves expressing BKT or PSY + BKT (*n* ≥ 3, **P < 0.01, *t*‐test, error bar: SD). (e) qRT‐PCR analysis of *ZmPSY1* and *CrBKT* expression in infiltrated tobacco leaves (*n* ≥ 3, **P < 0.01, *t*‐test, error bar: SD).

Referring to relevant studies in other plant species, we designed an astaxanthin biosynthesis strategy in cotton. Four key genes were selected to reconstruct the astaxanthin biosynthetic pathway: *ZmPSY1* from *Zea mays* (Zhu *et al*., 2008), *PaCrtI* from *Pantoea ananatis* (Misawa *et al*., 1990), *HpCrtZ* from *H. pluvialis* (Zhou *et al*., 2015), and *CrBKT* from *Chlamydomonas reinhardtii* (Lohr *et al*., 2005). Since heterologous gene expression in cotton plastids could be affected by differences in codon usage, the gene sequences of *PaCrtI, HpCrtZ*, and *CrBKT*, derived from algae and bacteria, were codon‐optimized for efficient expression in cotton (Figure S1). Subsequently, individual expression vectors for the four genes (designated as ‘X1’, Figure 2a) were constructed using the Cotton2.0 overexpression vector (Hu *et al*., 2021), with each gene fused to a rubisco transit peptide to ensure plastid localization.

Given the heavy workload and longtime procedure of stable cotton transformation, we first validated the constructs using an agroinfiltration‐based transient expression system in tobacco leaves. A total of 12 expression combinations were tested, including single‐gene expression with BKT, PSY, CrtI, and CrtZ. Dual‐gene co‐expression included BKT + PSY, BKT + CrtI, and BKT + CrtZ. Triple‐gene co‐expression was tested for BKT + PSY + CrtI, BKT + PSY + CrtZ, and BKT + CrtI + CrtZ. Additionally, a four‐gene co‐expression combination of BKT + PSY + CrtI + CrtZ was included. An empty vector (EV) control was used as a negative control. Regardless of whether a single gene or multiple genes were co‐expressed, constructs containing BKT consistently induced red pigmentation in tobacco leaves (Figure 2b,c). This red colouration is a characteristic marker of astaxanthin accumulation, indicating that the engineered pathway effectively altered carotenoid metabolism. To confirm astaxanthin biosynthesis, representative tobacco leaves expressing BKT alone and PSY + BKT were selected for high‐performance liquid chromatography (HPLC) analysis. The results demonstrated that these tobacco leaves synthesized significant amounts of astaxanthin, with 100.11 μg/g DW and 128.38 μg/g DW, respectively, whereas no detectable astaxanthin was observed in the control leaves infiltrated with the EV (Figure 2d). Additionally, qRT‐PCR analysis confirmed that *CrBKT* and *ZmPSY1* were highly expressed in tobacco leaves, verifying the functional expression of the engineered constructs (Figure 2e). These results suggest that the constructed vectors can be effectively applied for subsequent stable transformation in cotton.

### Overexpression of *CrBKT* produces astaxanthin in cotton callus and somatic embryos

**Figure 3:**
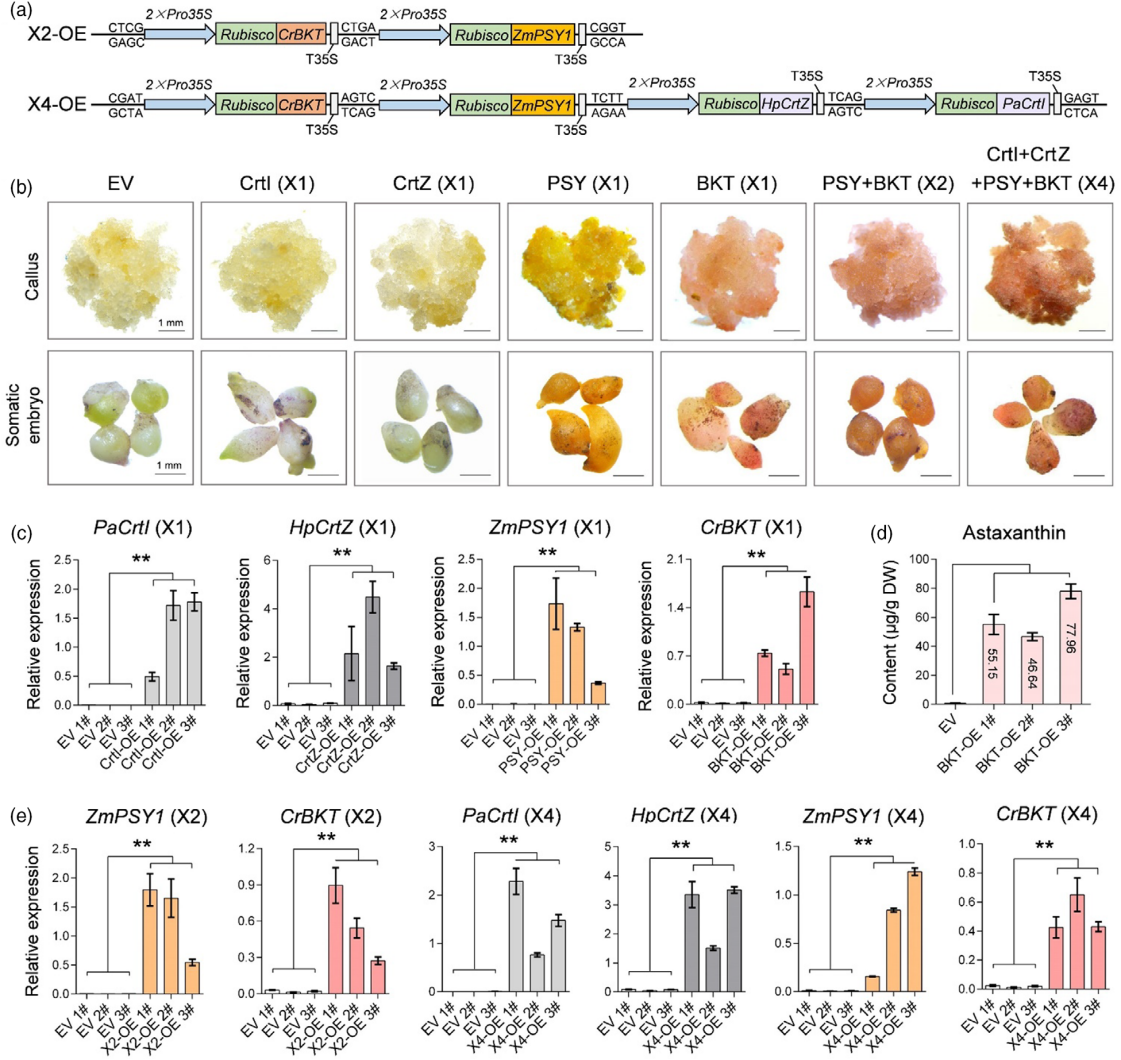
Stable expression of exogenous genes enables astaxanthin biosynthesis in cotton callus and embryogenic tissues. (a) Schematic representation of multi‐gene overexpression vectors: X2 (*CrBKT* + *ZmPSY1*) and X4 (*CrBKT* + *ZmPSY1* + *HpCrtZ* + *PaCrtI*) used for stable transformation in cotton. (b) Representative callus and embryogenic tissues from transgenic cotton. EV, empty vector. Scale bar, 1 mm. (c) qRT‐PCR analysis of exogenous gene expression in cotton callus transformed with X1. 1#, 2#, and 3# represent different transgenic lines (*n* ≥ 3, **P < 0.01, *t*‐test, error bar: SD). (d) HPLC analysis of astaxanthin content in transgenic cotton callus expressing *CrBKT* (*n* ≥ 3, **P < 0.01, *t*‐test, error bar: SD). (e) qRT‐PCR analysis of exogenous gene expression in cotton callus transformed with X2 and X4 (*n* ≥ 3, **P < 0.01, *t*‐test, error bar: SD).

To achieve astaxanthin biosynthesis in cotton, in addition to the four single‐gene expression vectors (X1) described earlier, we constructed multi‐gene expression cassettes. These included a dual‐gene expression vector carrying PSY and BKT (X2), as well as a four‐gene expression vector BKT + PSY + CrtI+CrtZ (X4) (Figure 3a). Along with the EV control, a total of seven vectors were introduced into cotton through *Agrobacterium*‐mediated stable transformation, using *Gossypium hirsutum* L. ‘Jin668’ as the explant material.

In transgenic callus with confirmed positive transformation, callus expressing BKT (X1), X2, and X4 exhibited a distinct red colouration, indicating astaxanthin accumulation. In contrast, the PSY (X1) callus displayed a remarkable yellow pigmentation, suggesting overaccumulation of carotenoids. Meanwhile, callus expressing CrtZ or CrtI exhibited no visible colour differences compared to the EV (Figure 3b). However, the red pigmentation in BKT (X1), X2, and X4 callus lines showed considerable variation among different transformation lines. Notably, the red colouration in X4‐positive callus was not necessarily more intense than in X2 or BKT (X1) alone. To further investigate these variations, qRT‐PCR analysis was performed on selected callus lines (Figure 3c,e). The results revealed that the expression levels of the target genes varied significantly between lines, correlating with the observed pigmentation differences. Some BKT (X1) or X2 callus lines exhibited higher *CrBKT* expression than certain X4 callus lines. These findings suggest that *CrBKT* expression is the key factor in astaxanthin biosynthesis in cotton, and although multi‐gene co‐expression theoretically enhances the metabolic flux toward astaxanthin production, the variation in gene expression among individual lines could exceed the differences between designed expression constructs. HPLC analysis confirmed that the pigmentation differences in transgenic callus were due to astaxanthin accumulation resulting from gene overexpression. In three representative BKT lines, astaxanthin content reached 46.64, 55.15, and 77.96 μg/g DW in callus, respectively (Figure 3d). These results demonstrate that expressing exogenous genes in cotton can successfully enable astaxanthin biosynthesis, establishing cotton as a viable platform for astaxanthin production.

During embryogenic development, distinct red pigmentation was still observed in certain BKT lines, including those transformed with X1, X2, and X4 (Figure 3b). However, no red colouration was detected in any CrtZ, CrtI, or PSY lines, although some PSY lines exhibited characteristic yellow or yellow‐orange pigmentation, indicative of carotenoid accumulation. Although all transformed cotton callus lines were able to regenerate plants, an undesirable phenomenon was observed. Cotton lines overexpressing *ZmPSY1*, including PSY (X1), X2, and X4, exhibited reduced regeneration efficiency, making it more difficult to obtain somatic embryos and regenerate viable plants. Furthermore, even when some regenerants were obtained, they tended to gradually die during the tissue culture stage (Figure S2). In contrast, BKT plants successfully continued their development. These findings indicate that *CrBKT* alone is sufficient for astaxanthin biosynthesis in cotton. However, constitutive overexpression of *ZmPSY1* significantly increased the metabolic flux toward carotenoid biosynthesis, which somehow exerted a negative impact on somatic embryo formation and plant regeneration in cotton.

### Stable expression and inheritance of astaxanthin in transgenic cotton plants

**Figure 4:**
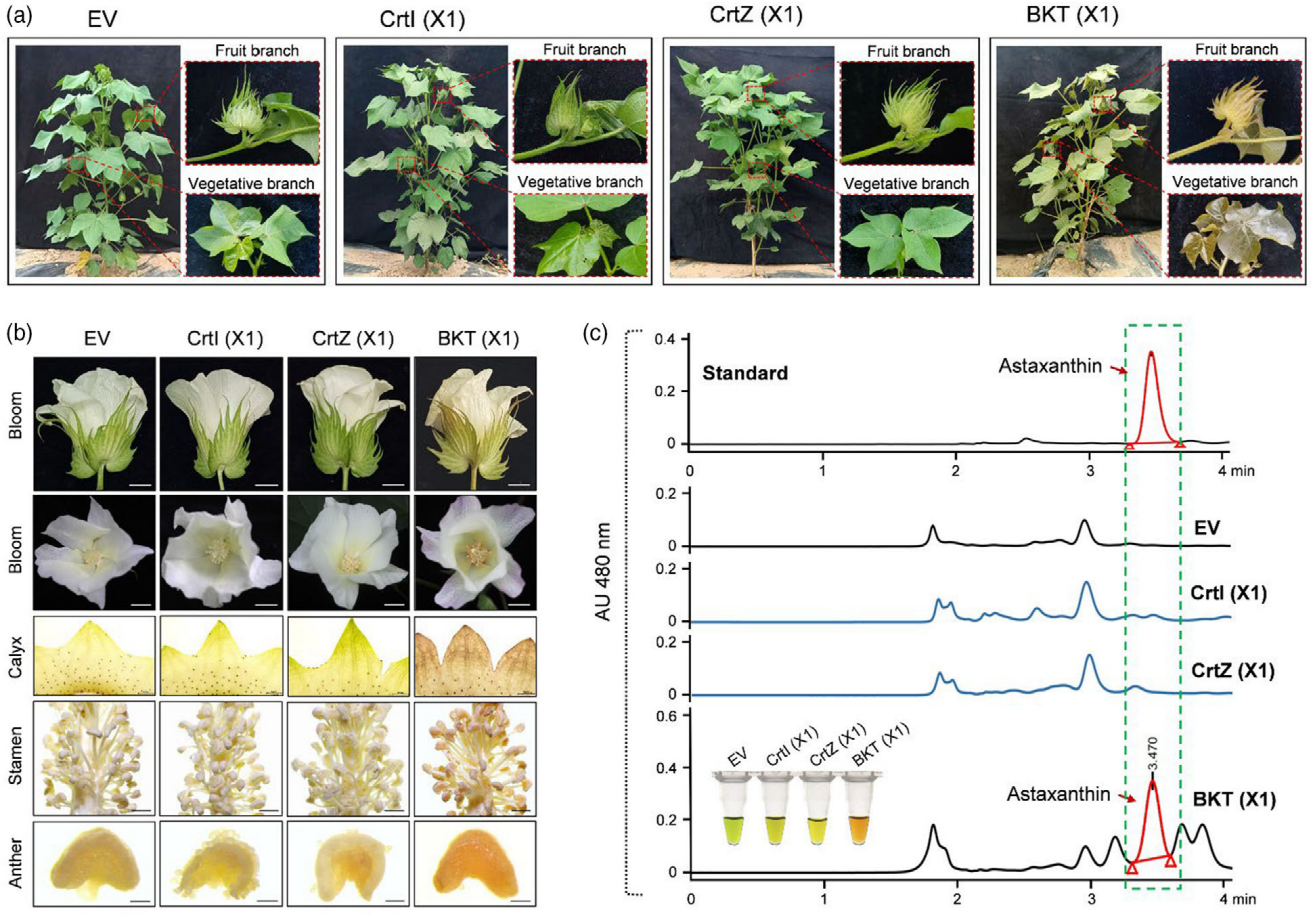
Astaxanthin production in transgenic cotton under field conditions. (a) Phenotypic characteristics of transgenic cotton plants at the reproductive stage, including whole plants, fruiting branches, and vegetative branches. EV, empty vector. (b) The BKT plant exhibits light‐red colouration in various floral organs, including bracts, calyxes, and stamens. Scale bar: 1 cm for blooms; 2 mm for calyx and stamen; 250 μm for anther. (c) HPLC analysis of astaxanthin content in leaf extracts of transgenic cotton. Red arrows indicate astaxanthin peaks. The leaf extracts are shown on the lower left.

Field observations revealed that, in contrast to EV, CrtI (X1) and CrtZ (X1) plants, the red pigmentation associated with astaxanthin accumulation was stably present in transgenic BKT cotton plants at multiple developmental stages. In vegetative organs, BKT (X1) plants exhibited a distinct red colouration throughout all green tissues, including leaves, petioles, stipules, and stems. This pigmentation was more prominent in young tissues and became less pronounced in mature, chlorophyll‐rich tissues, likely due to chlorophyll masking the red colouration (Figure 4a). In reproductive organs, red pigmentation was also observed in bracts, calyxes, and stamens (Figure 4b). Compared to other floral structures, petals exhibited relatively weak colour changes. Additionally, no red pigmentation was detected in mature cotton fibres. HPLC analysis confirmed the presence of astaxanthin in BKT plants. A characteristic peak, corresponding to endogenously synthesized astaxanthin, was identified in BKT samples (marked with red arrows), matching the retention time of the astaxanthin standard. In contrast, no astaxanthin peaks were observed in the HPLC profiles of EV, CrtI, or CrtZ plants (Figure 4c).

To investigate the stability and inheritance of *CrBKT* expression, self‐pollinated T_1_ lines were cultivated. The results demonstrated that T_1_ generation BKT plants displayed the characteristic red pigmentation, while EV plants exhibited no visible red colouration (Figure S3). Furthermore, no significant differences in growth or development were observed between EV and BKT T_1_ plants, indicating that the heterologous *CrBKT* gene and its associated astaxanthin accumulation were stably inherited by the next generation.

### Single *BKT* expression directs carotenoid metabolic flux towards astaxanthin in cotton

**Figure 5:**
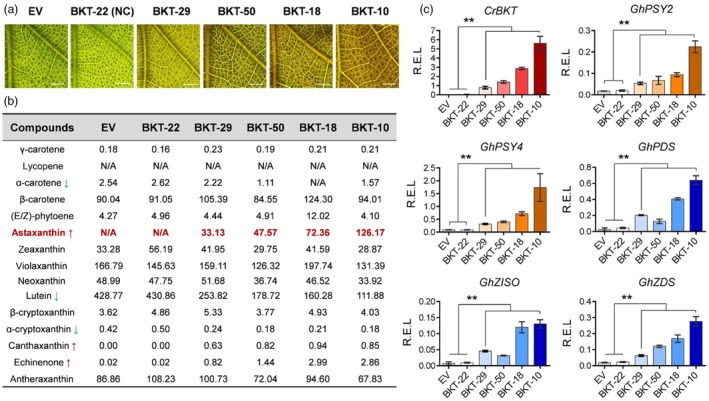
Correlation between red pigmentation, *CrBKT* expression, and astaxanthin accumulation in transgenic cotton. (a) Representative leaves of transgenic cotton plants showing red colouration. EV, empty vector; NC, negative control (BKT plants without *CrBKT* expression). Scale bar, 2 mm. (b) UPLC‐MS/MS analysis of carotenoid composition and content in transgenic cotton leaves. Red and green arrows indicate upregulated and downregulated metabolites, respectively, in BKT cotton. Metabolite content is presented as μg/g dry weight (DW). (c) qRT‐PCR analysis of *CrBKT* and endogenous carotenoid biosynthetic genes in EV, BKT‐22 (NC), and other BKT lines. R.E.L., relative expression level (*n* ≥ 3, **P < 0.01, *t*‐test, error bar: SD).

To investigate the role of BKT in regulating carotenoid metabolism and astaxanthin biosynthesis, we conducted qRT‐PCR and UPLC‐MS/MS analyses on leaves from different BKT transgenic lines exhibiting varying degrees of red pigmentation. The results revealed a positive correlation between the intensity of red colouration, *CrBKT* expression levels, and astaxanthin accumulation (Figure 5). In true leaves, no astaxanthin signals were detected in EV or BKT‐22, a transgenic line lacking *CrBKT* expression that was used as a negative control (NC). In contrast, four representative BKT lines (BKT‐29, BKT‐50, BKT‐18, and BKT‐10) exhibited progressively increased *CrBKT* expression and intensified red pigmentation (Figure 5a,c), with astaxanthin contents of 33.13, 47.57, 72.36, and 126.17 μg/g DW, respectively (Figure 5b).

Notably, in BKT‐10, astaxanthin accounted for 20.43% of total tested carotenoids, demonstrating the potential of cotton as a bioengineering platform for astaxanthin biosynthesis (Figure 5b). Additionally, *CrBKT* expression led to a significant increase in the astaxanthin precursors, canthaxanthin and echinenone. The canthaxanthin level increased to 0.94 μg/g DW, while echinenone rose from 0.02 to 2.99 μg/g DW. Conversely, a marked decrease was observed in an alternative metabolic branch involving α‐carotene, which had an initial concentration of 2.54 μg/g DW (2.92‰ of total carotenoids) in EV but significantly declined in BKT lines. The α‐carotene‐derived lutein, which accounted for 49.23% of total carotenoids in EV (428.77 μg/g DW) and 430.86 μg/g DW in BKT‐22 (NC), decreased to 111.88 μg/g DW in BKT‐OE cotton leaves. Similarly, α‐cryptoxanthin content declined from 0.42 μg/g DW in EV to 0.18 μg/g DW in BKT‐OE lines. These findings suggest that introducing *CrBKT* in cotton successfully redirects carotenoid metabolism toward astaxanthin biosynthesis, leading to a reduction in alternative branch metabolites such as α‐carotene, lutein, and α‐cryptoxanthin. The redistribution of metabolic resources further indicates that *CrBKT* expression successfully activates the astaxanthin biosynthetic pathway in cotton.

To further explore the transcriptional changes associated with carotenoid metabolism, we performed qRT‐PCR analysis on EV, NC, and BKT cotton leaves (Figure 5c). Compared to EV and NC leaves, BKT leaves exhibited significant upregulation of multiple genes involved in carotenoid biosynthesis. PSY is the rate‐limiting enzyme that controls terpene flux into the carotenoid biosynthetic pathway. Among the eight PSY homologues in cotton, two (*GhPSY2* and *GhPSY4*) were highly upregulated in BKT leaves, reaching expression levels dozens of times higher than in EV. In addition, homologues of CrtI in cotton, including *GhPDS*, *GhZISO*, and *GhZDS*, were also significantly upregulated in BKT plants. These transcriptional changes indicate that alterations in carotenoid metabolism due to *CrBKT* expression triggered feedback regulation at the transcriptional level.

### Overexpression of *CrBKT* produces larger cottonseeds

**Figure 6:**
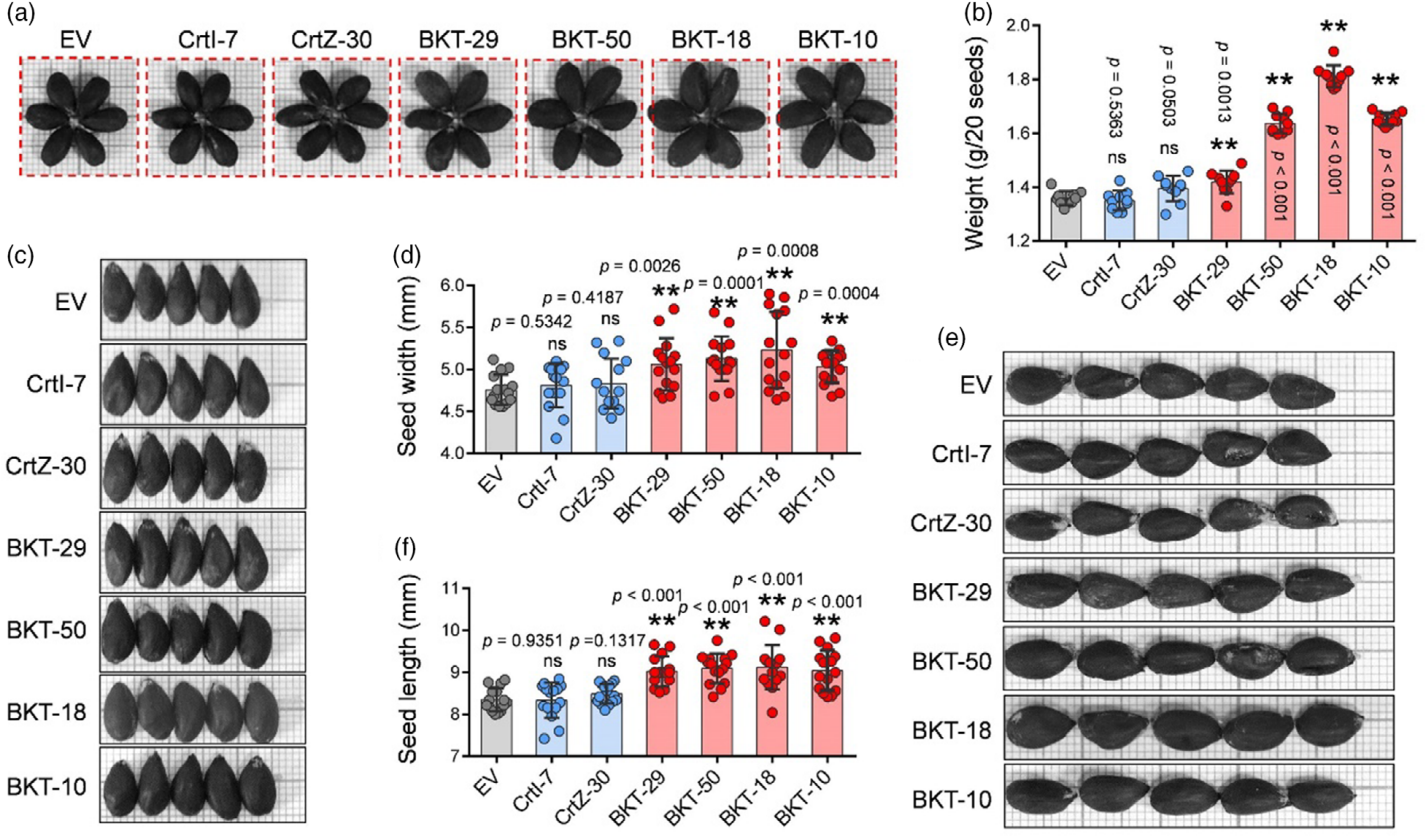
Overexpression of *CrBKT* increases cottonseed size. (a, c, e) Representative cottonseeds from EV, CrtI, CrtZ, and BKT lines. Scale bar: each grid represents 1 mm × 1 mm. (b, d, f) Statistical analysis of seed weight (b), seed width (d), and seed length (f) in transgenic cotton (*n* ≥ 8 for seed weight, *n* ≥ 15 for seed size, **P < 0.01, ns means no significance, *t*‐test, error bar: SD).

Interestingly, we found that *CrBKT*‐overexpression significantly altered cottonseed size (Figure 6a). To investigate this phenomenon, the seeds of BKT lines were compared with those of EV, CrtI, and CrtZ. The weight of cottonseeds was measured, revealing similar weights among EV (1.36 g/20 seeds), CrtI (1.36 g/20 seeds), and CrtZ (1.39 g/20 seeds), and the increased weight in BKT lines, with average seed weights of 1.42 g (BKT‐29), 1.64 g (BKT‐50), 1.81 g (BKT‐18), and 1.65 g (BKT‐10) per 20 seeds (Figure 6b). Statistical analysis confirmed no significant differences in seed weight among EV, CrtI, and CrtZ, whereas BKT lines exhibited a significant increase in seed weight compared to all three control groups (Figure 6b). Further seed size measurements were conducted using a vernier calliper. The average seed width increased from 4.76 mm in EV to a range of 5.03–5.23 mm in BKT lines. In contrast, the average seed width in CrtI and CrtZ was 4.81 and 4.83 mm, respectively (Figure 6c,d). A significant increase in seed length was also observed. The seed length was 8.34 mm in EV, 8.33 mm in CrtI, and 8.49 mm in CrtZ, while in BKT lines, the seed length ranged from 9.02 to 9.12 mm (Figure 6e,f). These results suggest that the overexpression of *CrBKT* also contributes to the production of larger cottonseeds.

### Metabolic shifts in BKT cottonseeds with enhanced astaxanthin biosynthesis and reduced gossypol accumulation

**Figure 7:**
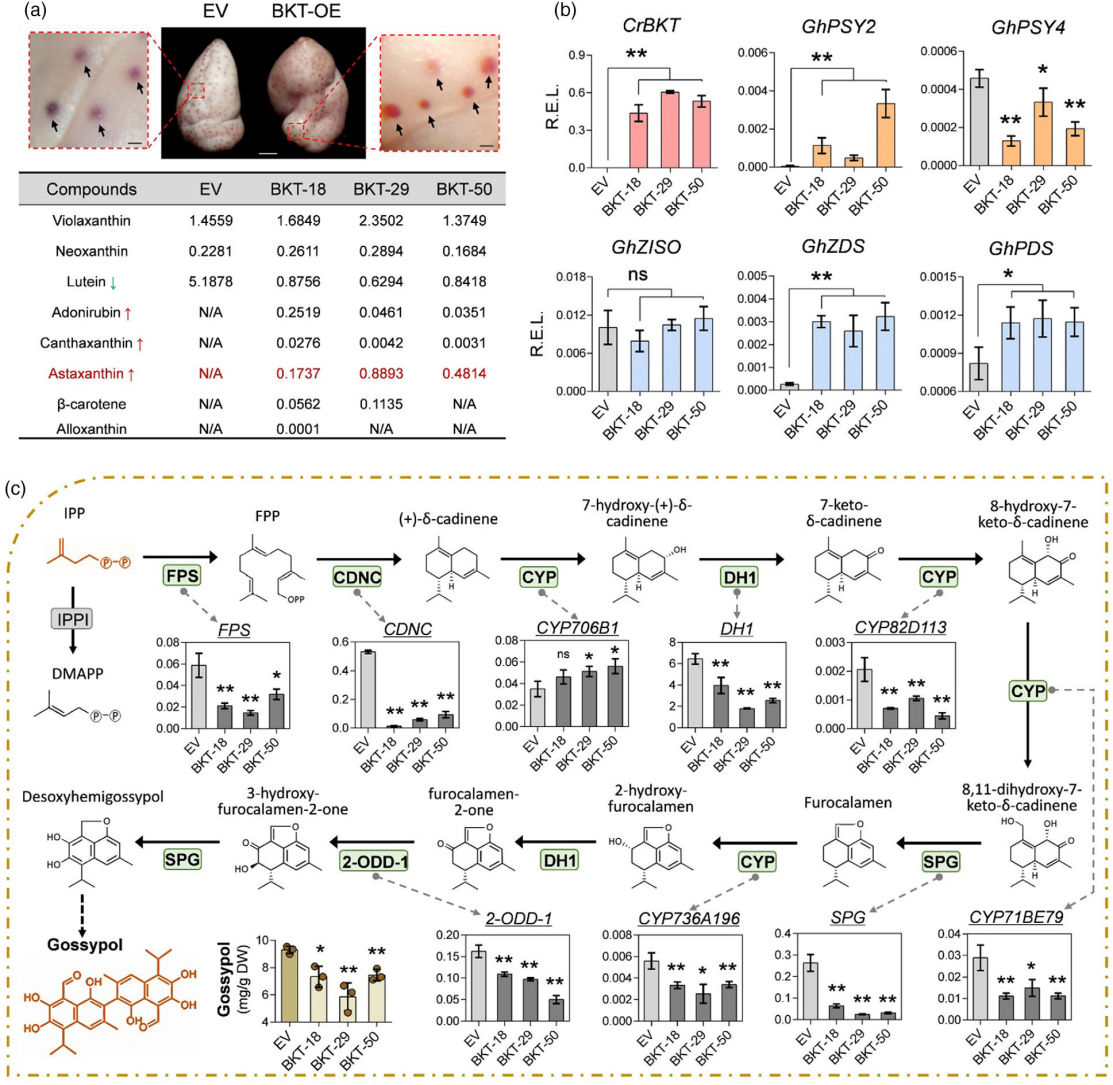
Metabolic analysis of carotenoid and gossypol biosynthesis in transgenic cottonseeds. (a) Representative images of cottonseed kernels from EV and BKT‐OE and UPLC‐MS/MS analysis of carotenoid content in cottonseed kernels. Pigment glands are marked with black arrows. Scale bar: 1 mm for cottonseeds, 100 μm for enlarged pigment glands. Red and green arrows indicate upregulated and downregulated metabolites, respectively, in BKT cottonseeds. Metabolite content is presented as μg/g dry weight (DW). (b) qRT‐PCR analysis of *CrBKT* and endogenous carotenoid biosynthetic genes in cottonseed kernels (*n* ≥ 3, *P < 0.05; **P < 0.01, ns means no significance, *t*‐test, error bar: SD). (c) Schematic representation of the gossypol biosynthetic pathway in cotton. Relative gene expression levels were analyzed by qRT‐PCR, and the Y‐axis represents the relative gene expression level. The gossypol content in cottonseed kernels was quantified by HPLC (*n* ≥ 3, *P < 0.05; **P < 0.01, ns means no significance, *t*‐test, error bar: SD).

In addition to the increase in seed size, BKT cottonseeds exhibited uneven light‐red pigmentation in the kernel, whereas EV seeds maintained a creamy colour (Figure 7a). UPLC‐MS/MS analysis revealed that the total carotenoid content in seeds was relatively low compared to leaves and other tissues. However, astaxanthin was successfully detected in BKT seeds, whereas no astaxanthin was detected in EV seeds (Figure 7a). Similar to the findings in leaves, multiple genes involved in carotenoid biosynthesis showed a certain degree of upregulation in BKT seeds (Figure 7b).

The pigment glands in cottonseeds are the sites for secondary metabolite synthesis and accumulation. We observed a dramatic colour change in the pigment glands of BKT cotton, shifting from dark brown to red (Figure 7a), suggesting a potential alteration in secondary metabolite composition. Gossypol, a lipophilic sesquiterpene compound, is one of the major secondary metabolites that accumulate in cottonseeds (Tian *et al*., 2018). Its biosynthesis originates from IPP, which shares a common upstream metabolic precursor with astaxanthin biosynthesis. To investigate the impact of *CrBKT* overexpression, we quantified gossypol content in BKT seeds. HPLC analysis showed that gossypol content in BKT seeds was reduced by 19.9–37.2% compared to the EV seeds (Figure 7c). Consistently, genes involved in gossypol biosynthesis exhibited significant transcriptional changes. qRT‐PCR analysis revealed that, except for *CYP706B1*, most gossypol biosynthetic genes were downregulated in astaxanthin‐accumulating cottonseeds (Figure 7c). These included *FPS*, which encodes a key enzyme directing IPP toward gossypol biosynthesis, and *CDNC*, which encodes the rate‐limiting enzyme in the gossypol biosynthetic pathway. Several well‐characterized gossypol biosynthetic genes, whose expression has been reported to be closely related to gossypol synthesis in pigment glands, such as *GhCYP82D113*, *GhSPG*, and *Gh2‐ODD‐1*, were significantly downregulated in BKT seeds. Additionally, BKT cotton leaves also exhibited a moderate downregulation of gossypol‐related genes (Figure S4), further supporting the notion that enhancing astaxanthin biosynthesis can suppress gossypol biosynthesis to a certain extent.

## Discussion

### Cotton as a promising platform for natural astaxanthin production

Cotton is one of the world's most widely cultivated crops, with extensive geographical distribution and significant economic value (Huang *et al*., 2021). Its broad cultivation area and adaptability make it an attractive candidate for astaxanthin production, particularly considering that cotton's vegetative organs, such as leaves, currently have no commercial use and require additional labour for pruning (Ding *et al*., 2024; Liu *et al*., 2018). Our results confirm that cotton leaves can serve as an efficient biofactory for large‐scale astaxanthin biosynthesis, highlighting its potential as a sustainable platform for carotenoid production. By leveraging cotton's established agricultural infrastructure, production costs could be reduced while taking advantage of the crop's extensive cultivation base. This is in stark contrast to microalgae‐based astaxanthin production, which requires specialized growth conditions and complex harvesting techniques (Miao *et al*., 2023). Notably, while astaxanthin biosynthesis has been engineered in crops like tobacco (up to 717 μg/g DW in leaves; Wang *et al*., 2025), tomato (16.1 mg/g in fruits; Huang *et al*., 2013), and maize (111.82 mg/kg DW in seeds; Liu *et al*., 2021), these systems often rely on multigene transformations or tissue‐specific promoters. In contrast, cotton achieves comparable astaxanthin yields (126.17 μg/g DW in leaves) through single‐gene *CrBKT* overexpression, avoiding the metabolic burden and technical complexity. Thus, cotton‐based production provides a cost‐effective and scalable alternative for astaxanthin biosynthesis.

This study further demonstrates that *CrBKT* expression alone is sufficient to drive astaxanthin biosynthesis in multiple cotton tissues, including leaves, stems, and reproductive organs. This suggests that cotton's existing terpenoid metabolic framework—rich in hydroxylases and ketolases—facilitates astaxanthin production and potentially the biosynthesis of other bioactive terpenoids, such as artemisinin and paclitaxel, making its biotechnological application more feasible (Lange, 2015; Lommen *et al*., 2006). For instance, engineered tobacco and rice often require co‐expression of *CrtZ* and *CrtW* to optimize ketocarotenoid flux (Hasunuma *et al*., 2008; Mortimer *et al*., 2017; Zhu *et al*., 2018), whereas cotton's endogenous hydroxylases efficiently support BKT‐mediated conversion of β‐carotene to astaxanthin. More importantly, *CrBKT* expression alone offers a simple and efficient metabolic engineering strategy for astaxanthin biosynthesis in cotton without requiring complex multi‐gene transformations, thereby simplifying genetic modification processes and overcoming challenges associated with multi‐gene expression in transgenic plants (Schiemann *et al*., 2019).

### Astaxanthin synthesis in cottonseeds competes with gossypol biosynthesis

Cottonseed, with its high content of edible oil (21%) and protein (23%), possesses significant nutritional potential. Globally, for every kilogram of cotton fibre produced, approximately 1.6 kilograms of cottonseed are generated (Lambou *et al*., 1966; Sunilkumar *et al*., 2006). With an annual global production of ~40 million tons, cottonseed represents a valuable but underutilized food source. However, cotton's pigment glands, which serve as the primary sites for secondary metabolite biosynthesis (Long *et al*., 2025; Zhang *et al*., 2024), accumulate gossypol and its derivatives, rendering cottonseed toxic to humans and many animals, thereby limiting its potential as a food and feed ingredient (Brimer and Sørensen, 2009).

For decades, breeders have attempted to eliminate gossypol toxicity to increase cottonseed's commercial value (Sunilkumar *et al*., 2006). Recent studies have demonstrated that the innermost secretory cells of pigment glands produce gossypol, with key biosynthetic genes expressed at levels hundreds to thousands of times higher than in other cells. Additionally, the upstream genes, including *HMGS* and *PMD* in the MVA pathway, are also highly expressed in gland cells, supplying precursors for both gossypol and astaxanthin biosynthesis (Long *et al*., 2023; Zhang *et al*., 2024). Unlike other crops, where the kernel primarily supports protein and oil synthesis, cottonseeds contain specialized pigment glands, providing a distinct metabolic compartment for astaxanthin biosynthesis.

In rice, researchers successfully established a *de novo* astaxanthin biosynthetic pathway in endosperm by simultaneous expression of four genes (*ZmPSY1*, *PaCrtI*, *CrBKT*, and *HpBHY*) under endosperm‐specific promoters (Zhu *et al*., 2018). Similarly, astaxanthin‐enriched maize kernels were generated by co‐expressing *PaCrtI*, *ZmPSY1*, *CrBKT*, and *HpCrtZ* (Liu *et al*., 2021). This study shows that the single expression of *CrBKT* in cottonseeds competes with gossypol biosynthesis, as astaxanthin production redirects metabolic flux away from gossypol biosynthesis. Both gossypol and astaxanthin biosynthesis share a common precursor IPP. IPP is directed toward gossypol synthesis via FPS or toward astaxanthin biosynthesis via GGPPS, and possibly to other terpenoids (Lu and Li, 2018; Tian *et al*., 2018). Enhancing astaxanthin biosynthesis leads to metabolic resource reallocation, resulting in reduced gossypol accumulation in cottonseeds. This shift is further supported by the downregulation of key gossypol biosynthetic genes (*CNDC*, *DH1*, *CYP82D113*, *SPG*, *2‐ODD‐1*, and others), demonstrating that introducing *CrBKT* not only redirects metabolic flux but also alters the transcriptional regulation of secondary metabolism. The significant colour change observed in the pigment glands of BKT cottonseeds provides additional evidence that *CrBKT* expression modifies secondary metabolite accumulation patterns.

Cottonseed oil contains approximately 14% oleic acid and 59% linoleic acid. Increasing oleic acid content enhances oxidative stability and nutritional value (Chen *et al*., 2021). As China's demand for edible oil continues to rise, improving cottonseed oil quality has become one of the major focuses of cottonseed utilization (Li *et al*., 2024). Like gossypol, astaxanthin is lipid‐soluble, suggesting that replacing gossypol with astaxanthin in cottonseed may improve the oxidative stability of cottonseed oil. In summary, converting gossypol into astaxanthin not only reduces toxicity but also enhances the functional and nutritional value of cottonseed, making it a more suitable ingredient for human consumption and livestock feed. This metabolic transformation provides dual benefits: improving nutritional value while eliminating toxicity constraints.

### Astaxanthin accumulation increases cottonseed size likely due to the redistribution of terpenoid metabolic pathways

One of the most remarkable findings of this study is the significant increase in seed size and weight in *CrBKT*‐overexpressing cotton lines. This provides additional economic incentives for incorporating astaxanthin biosynthesis into cotton breeding programs. The correlation between *CrBKT* expression and increased seed biomass suggests that astaxanthin biosynthesis may influence seed development and metabolic resource allocation.

Although the exact mechanism remains unclear, we hypothesize that astaxanthin and its precursors accumulate in the cotton kernel, leading to metabolic shifts that enhance nutrient partitioning for seed growth. Additionally, alteration in terpenoid metabolism could affect the biosynthesis of terpenoid plant hormones, such as gibberellin acid (GA), strigolactone (SL), and abscisic acid (Pichersky and Raguso, 2018). The biosynthesis of GAs and gossypol shares the upstream MVA pathway. GGPP serves as a precursor for GA biosynthesis and enters the GA synthesis pathway by ent‐copalyl diphosphate synthase (Hedden, 2020). In a recent study, it was found that knocking out *GhHAM*, a gland‐forming gene, similarly affected gossypol synthesis and GA production, suggesting a correlation between gossypol and GA in glands (Long *et al*., 2024). Furthermore, in rice, GA_4_ regulates grain width by modulating glume cell size (Dang *et al*., 2024). In maize, the SL network has been reported to coordinate grain size evolution with kernel‐bearing cupule architecture (Guan *et al*., 2022). This complex interplay between terpenoid metabolism, hormonal regulation, and seed development highlights the need for further research to elucidate the precise molecular mechanisms underlying seed growth modulation.

## Conclusion

This study highlights the successful establishment of an astaxanthin biosynthetic pathway in cotton through single‐gene expression of *CrBKT*, demonstrating its feasibility for large‐scale production. In addition to astaxanthin accumulation, *CrBKT* expression led to larger seeds and a reduction in gossypol content, all of which have significant implications for agricultural, industrial, and nutritional applications. These findings provide a foundation for future research aimed at optimizing cotton as a biofactory for high‐value carotenoids and functional food ingredients. Further studies could focus on improving astaxanthin yields, understanding the molecular mechanisms underlying metabolic shifts, and exploring potential synergies with other bioengineering approaches. The successful implementation of this strategy in cotton paves the way for sustainable, plant‐based astaxanthin production while enhancing the economic and nutritional value of cottonseed.

## Materials and methods

### Expression analysis of endogenous carotenoid biosynthetic genes in cotton

According to the report by Yao *et al*. (2018), homologous sequences of carotenoid biosynthetic genes were identified in the *Gossypium hirsutum* genome (Zhang *et al*., 2015). The Cotton Multi‐Omics Database (CottonMD) (https://yanglab.hzau.edu.cn/CottonMD.1) was used to analyse transcriptome data, and the expression Heatmap module of TBtools (Chen *et al*., 2020) was employed to integrate RNA‐seq data from different tissues, including roots, stems, leaves, ovules, and developing seeds, to generate a heatmap illustrating the tissue‐specific expression patterns of carotenoid biosynthetic genes in cotton.

### Synthesis of heterologous genes and construction of expression vectors

To enhance expression efficiency in the host plant, codon optimization and gene synthesis were performed for *CrBKT* from *C. reinhardtii* (GenBank: MG976837), *HpCrtZ* from *H. pluvialis* (GenBank: AY187011.1), and *PaCrtI* from *P. ananatis* (GenBank: MG992314). The *ZmPSY1* gene from maize (GenBank: MG992315) was amplified from maize cDNA.

For the first round of single‐gene vector construction, a homologous recombination method was employed to fuse each gene with a Rubisco transit peptide from tobacco (Pinck *et al*., 1986). The enhanced constitutive 2 × 35S promoter was used to drive the expression of the Rubisco transit peptide and target gene, and the constructs were inserted into the Cotton2.0 vector (Hu *et al*., 2021). This resulted in the construction of four single‐gene expression vectors (X1): Cotton2.0‐BKT, Cotton2.0‐PSY, Cotton2.0‐CrtI, and Cotton2.0‐CrtZ.

For multi‐gene vector construction, the Golden Gate cloning method (Engler *et al*., 2009) was applied. Based on the first‐round single‐gene constructs, primers with *Bsa*I restriction sites were used to amplify each gene's expression cassette, which included the 2 × 35S promoter, Rubisco transit peptide, target gene, and T35S terminator. These expression cassettes were assembled into the P35Ng vector (Ma *et al*., 2015) to generate the X2 construct (P35Ng‐BKT + PSY). Additionally, BKT, PSY, CrtZ, and CrtI expression cassettes were inserted into the PBI121‐gg vector (Jiang *et al*., 2024) to generate the X4 construct (PBI121‐BKT + PSY + CrtZ+CrtI).

### Transient expression in tobacco

Tobacco plants (6–7 weeks old) with vigorous growth were selected for transient expression experiments (Hu *et al*., 2021). The GV3101 *Agrobacterium* strain carrying the target vectors was activated and cultured to an OD_600_ of 1.2–1.6. The bacterial culture was then centrifuged at 4000 rpm for 15 min, and the pellet was resuspended in an infiltration buffer (10 mM MgCl_2_, 10 mM MES, and 20 μM acetosyringone) to an OD_600_ of 0.6–0.8, followed by incubation at room temperature for 3 h. The resuspended *Agrobacterium* solution was infiltrated into tobacco leaves using a syringe, and the plants were incubated in the dark for 12 h, followed by 2–3 days of cultivation under 16 h light/8 h dark conditions for phenotypic analysis and further experiments. For multi‐gene co‐expression, *Agrobacterium* suspensions carrying different target vectors were mixed in equal proportions before infiltration.

### Cotton genetic transformation

The constructed expression vectors were electroporated into *Agrobacterium* strain ‘LBA4404’ and subsequently used for *Agrobacterium*‐mediated transformation of hypocotyls of *G. hirsutum* L. ‘Jin668’ (Zhu *et al*., 2023). Cotton transformation followed the protocol described by Jin *et al*. (2006). After transformation, the explants were cultured on a selection medium containing 50 μg/mL kanamycin, allowing for the selection of transgenic callus. The callus was then transferred to an induction medium to promote the development of embryogenic callus and transgenic plant regeneration. The growth of transgenic callus was monitored, and qRT‐PCR screening was performed to confirm the successful integration of target genes. Positive T_0_ transgenic seedlings were identified and were transplanted into a field or greenhouse for further growth and seed propagation. Subsequent phenotypic assessments and analyses were conducted on the transgenic cotton materials.

### Total RNA extraction and qRT‐PCR analysis

Total RNA was extracted from tobacco leaves (3 days post‐infiltration), T_0_ transgenic cotton callus, and young cotton leaves using the RNAprep Pure Plant Plus kit (TianGen, Beijing, China). cDNA synthesis was performed using HiScript® III 1st Strand cDNA Synthesis Kit (Vazyme, Nanjing, China), and the resulting cDNA was diluted 50‐fold for use as a template in qRT‐PCR analysis.

qRT‐PCR was conducted using a ChamQ SYBR Colour qPCR Master Mix (Vazyme Nanjing, China), with *GhUBQ7* (GenBank: DQ116441) as the internal reference gene for cotton. The relative expression levels of target genes were calculated using the $2^{-\Delta C_{t}}$ method. For tobacco qRT‐PCR analysis, the Actin gene (GenBank: NM_001425946.1) was used as the reference gene.

### Gossypol content determination in cottonseeds

The gossypol extraction and quantification process was performed according to the method described by Zhang *et al*. (2024).

### Astaxanthin extraction and HPLC quantification

Samples for astaxanthin extraction were freeze‐dried and ground into a fine powder using liquid nitrogen. A total of 100 mg of powdered sample was placed in a 10 mL centrifuge tube and extracted with 3 mL of hexane/acetone/ethanol (2:1:1, v/v/v). The mixture was sonicated for 30 min, with intermittent mixing every 5 min, while crushed ice was used to prevent overheating. After centrifugation at 8000 rpm for 10 min at 4 °C, the supernatant was collected, and the pellet was re‐extracted following the same procedure. The combined supernatants were filtered through a 0.22 μm microfiltration membrane, dried using nitrogen gas, and redissolved in 100 μL of methanol before HPLC analysis.

HPLC was performed on a WATERS ALLIANCE e2695 system using a 15 cm VP‐ODS C18 (5‐μm) column. The mobile phase consisted of methanol/dichloromethane/water/acetonitrile (85:5:5:5, v/v/v/v) at a flow rate of 0.8 mL/min (Zhu *et al*., 2018). Astaxanthin standard (CAS: 472‐61‐7; Solarbio, Beijing, China) was dissolved in methanol at concentrations of 0, 30, 60, 90, 120, and 150 μg/mL for standard curve generation.

### Carotenoid extraction and UPLC‐MS/MS analysis

Freeze‐dried samples were milled at 30 Hz for 1 min and extracted with 0.5 mL of hexane/acetone/ethanol (1:1:1, v/v/v) containing 0.01% BHT. The mixture was vortexed for 20 min at room temperature, centrifuged at 12 000 rpm for 5 min at 4 °C, and the supernatant was collected. The extraction was repeated, and the combined extracts were dried, redissolved in 150 μL dichloromethane, and filtered through a 0.22 μm microfiltration membrane (Inbaraj *et al*., 2008). Carotenoid profiling was conducted using a QTRAP 6500+ mass spectrometer (MS) coupled with an ExionLC™ AD UPLC system at Metware Biotechnology (Wuhan, China).

## Conflict of interest

The authors declare no conflict of interest.

## Author contributions

L.L., W.G., and S.X.J. designed the research. L.L. and Y.C.T. wrote the manuscript. Y.C.T., Z.N.Z., L.L., T.W.L., G.Y.H., S.Z.S., M.J., Z.P.X., G.Y.W., Y.B.F., Y.M.M., and H.G.S. performed the experiments and data analysis.

## Supporting information


**Figure S1** Comparison of the original and codon‐optimized sequences of *PaCrtI*, *HpCrtZ*, and *CrBKT*.
**Figure S2** Regeneration of transgenic cotton seedlings and observed developmental abnormalities.
**Figure S3** Astaxanthin production in T_1_ progeny of *CrBKT*‐overexpressing cotton.
**Figure S4** Relative expression of gossypol biosynthetic genes in cotton leaves.

## Data Availability

The data that support the findings of this study are available on request from the corresponding author.
