# Supplementary material for: Breeding triple‐advantage cottonseed with higher yield, enhanced nutrition, and reduced toxicity by redirecting terpenoid metabolism to astaxanthin
Source: Plant Biotechnol J. 2025 Apr 29;23(7):2839–51. doi: 10.1111/pbi.70116 (PMC12205890; doi:10.1111/pbi.70116)
Supplement: Supplementary file 1 — Figure S1 Comparison of the original and codon‐optimized sequences of PaCrtI, HpCrtZ, and CrBKT. Figure S2 Regeneration of transgenic cotton seedlings and observed developmental abnormalities. Figure S3 Astaxanthin production in T1 progeny of CrBKT‐overexpressing cotton. Figure S4 Relative expression of gossypol biosynthetic genes in cotton leaves. [file PBI-23-2839-s001.docx]

**Supplementary Figures and Figure Legends**
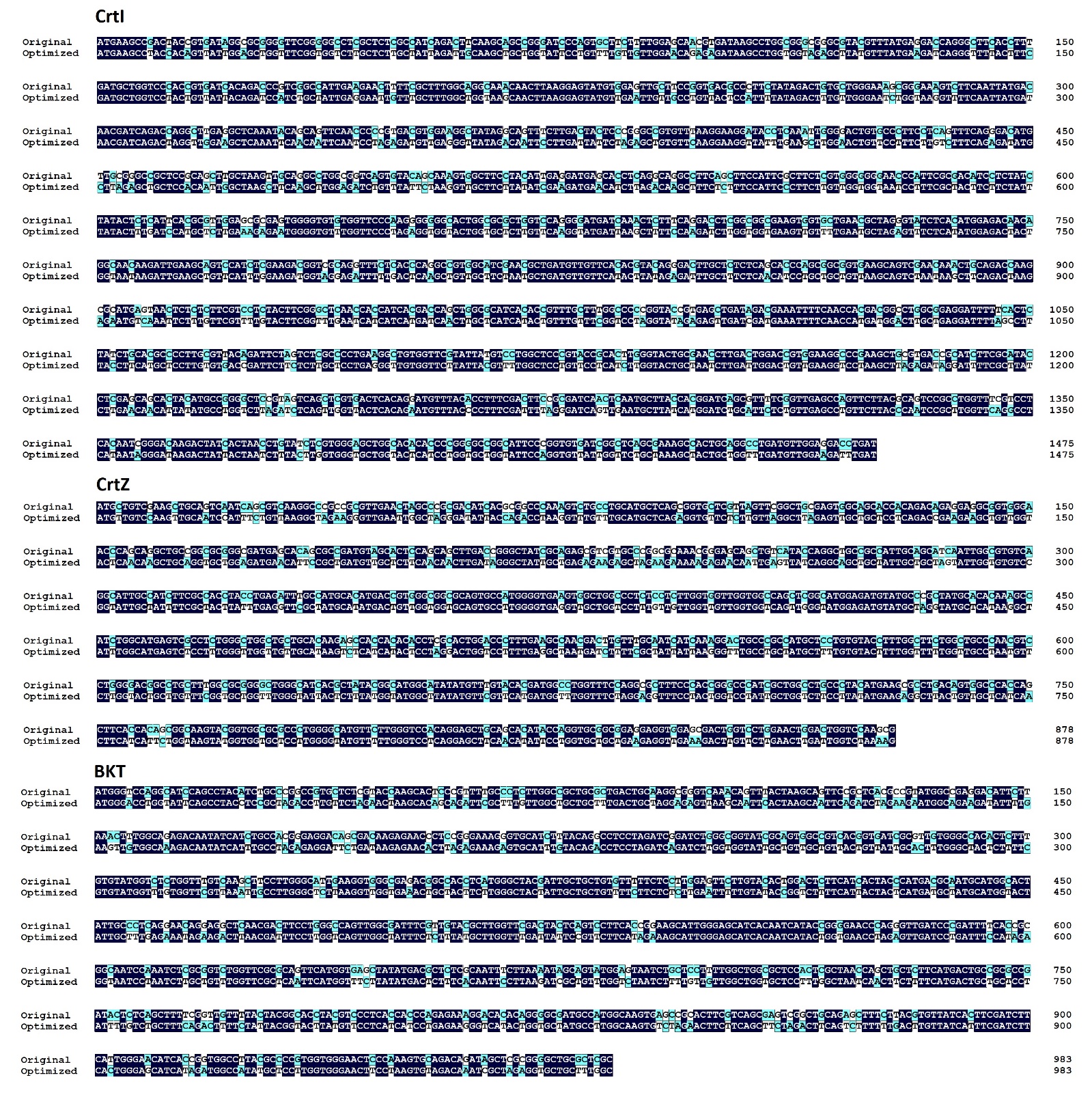


**Figure S1 Comparison of the original and codon-optimized sequences of *PaCrtI*, *HpCrtZ*, and *CrBKT*.**

Codon optimization was performed to enhance expression efficiency in cotton by improving codon usage compatibility with the cotton genome.

**
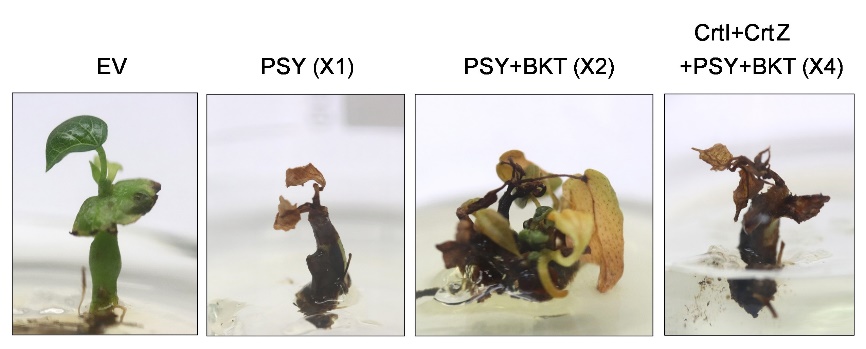
**

**Figure S2 Regeneration of transgenic cotton seedlings and observed developmental abnormalities.**

Representative images of regenerated seedlings from transgenic cotton expressing PSY (X1), PSY+BKT (X2), and PSY+BKT+CrtI+CrtZ (X4), showing signs of spontaneous seedling death. EV represents empty vector-transformed cotton plants as a control.

**
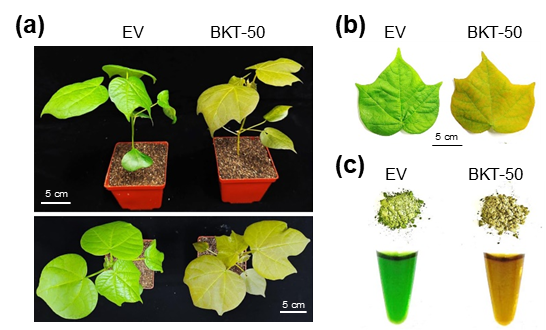
**

**Figure S3 Astaxanthin production in T_1_ progeny of *CrBKT*-overexpressing cotton.**

**(a)** Representative morphology of T_1_ progeny plants. Scale bar, 5 cm.

**(b)** True leaf phenotypes of EV and BKT-50 in the T_1_ generation, showing distinct pigmentation in transgenic plants.

**(c)** Comparison of leaf dry powder and pigment extracts from EV and BKT-50 true leaves, highlighting astaxanthin accumulation in transgenic lines.


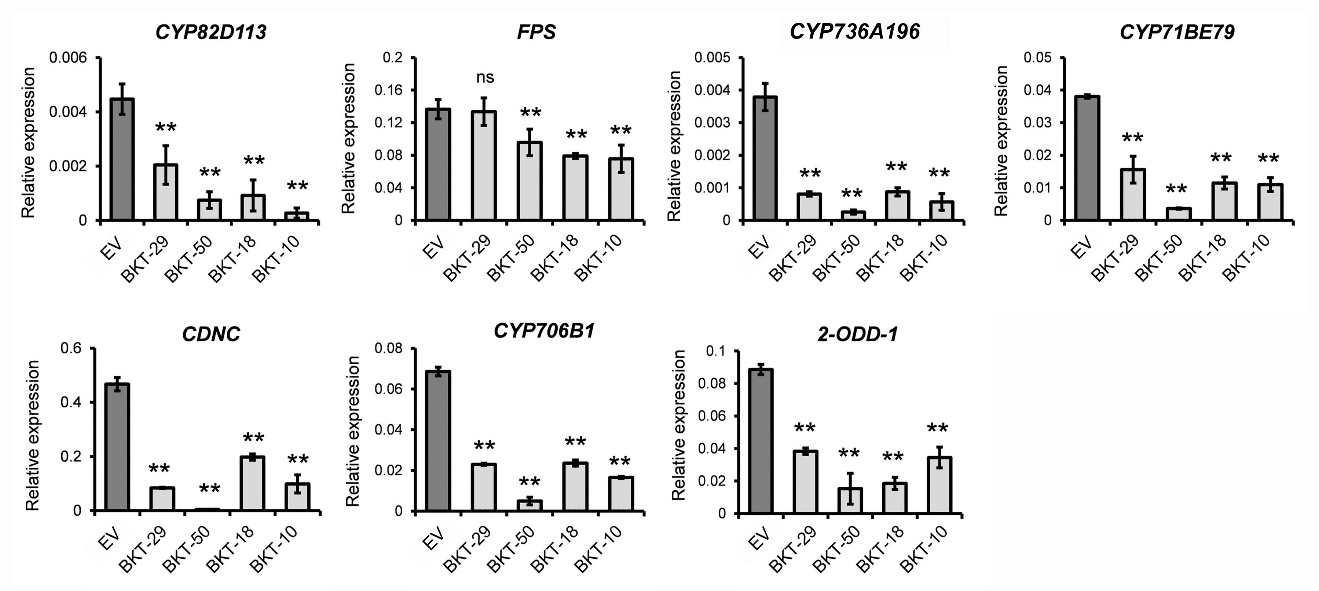


**Figure S4 Relative expression of gossypol biosynthetic genes in cotton leaves.**

qRT-PCR analysis of key genes involved in gossypol biosynthesis in EV and BKT-OE cotton leaves. (n ≥ 3, **P* < 0.05; ***P* < 0.01, ns means no significance, *t*-test, error bar: SD).
